# Sepsis-Induced Adipokine Change with regard to Insulin Resistance

**DOI:** 10.1155/2012/972368

**Published:** 2012-01-09

**Authors:** Andreas Hillenbrand, Manfred Weiss, Uwe Knippschild, Anna Maria Wolf, Markus Huber-Lang

**Affiliations:** ^1^Department of General, Visceral, and Transplantation Surgery, University Hospital of Ulm, Steinhoevelstraße 9, 89075 Ulm, Germany; ^2^Clinic of Anesthesiology, University Hospital of Ulm, Steinhoevelstraße 9, 89075 Ulm, Germany; ^3^Department of Traumatology, Hand and Reconstructive Surgery, University Hospital of Ulm, Steinhoevelstraße 9, 89075 Ulm, Germany

## Abstract

*Background*. Assessment of white adipose tissue has changed in recent years, with WAT now being considered as an active endocrine organ, secreting a large number of bioactive mediators, so-called adipokines. Besides other functions, these adipokines are involved in inflammatory response thereby exhibiting predominantly proinflammatory or anti-inflammatory properties and contribute to insulin resistance. *Methods*. Comprehensive review of the literature of the role of adipokines relevant to critical care medicine using PubMed search. *Results*. Adiponectin—the prototype of an anti-inflammatory and insulin-sensitizing adipokine—is diminished in sepsis, while resistin—a protein with proinflammatory properties—is elevated. Plasminogen activator inhibitor-1, interleukin (IL)-1, IL-6, IL-8, and IL-10, and tumor-necrosis-factor-alpha mediate insulin resistance and are elevated in sepsis, while retinol-binding protein-4 concentrations are significantly reduced in sepsis. Chemerin displays potent anti-inflammatory and insulin-resistance properties, while monocyte chemotactic protein-1—increased in sepsis—contributes to macrophage infiltration in adipose tissue and insulin resistance. *Conclusions*. The expression of adipokines in humans is altered as well in obese as in septic patients with elevated levels of proinflammatory adipokines. Changes in adipokine levels in acute sepsis could contribute to insulin resistance. Consequently, in critically ill patients, these alterations underline a possible contribution of adipokines in the development of hyperglycemia.

## 1. Introduction

Severe sepsis is a syndrome characterized by systemic inflammation and acute organ dysfunction in response to infection. This inflammatory response is largely mediated by pro- and anti-inflammatory cytokines, which are released into the systemic circulation. While these cytokines are a prerequisite to fight infection, their overzealous production is deleterious. White adipose tissue (WAT) is involved in biosynthesis of these cytokines. WAT was traditionally considered a long-term energy storage depot with few interesting attributes. Due to the dramatic rise in obesity and its secondary diseases like metabolic syndrome during the past decades, adipose tissue is now considered to be an active endocrine organ that releases a large number of bioactive mediators, so-called adipokines. So far, more than 50 adipokines are known, and their number is still rising (adipokines here defined as signaling proteinaceous factors mainly produced or released by adipose tissue) [1].

At the cellular level, there is a substantial heterogeneity in WAT, especially in obese individuals—with mature adipocytes accounting for only about half of the total cell content. Other cells in WAT include fibroblasts, endothelial cells, preadipocytes, and in particular macrophages residing in the stromavascular fraction of WAT [2]. By acting as transmitters of endocrine or paracrine signals, the secreted adipokines are involved in the inflammatory process and insulin resistance. The expression of these adipokines is altered in obese as well as in septic patients with elevated levels of proinflammatory adipokines [3]. Therefore, obesity is associated with the appearance of a chronic, low inflammatory state [4]. Insulin resistance is a common feature in obesity. In analogy, changes in adipokine levels in acute sepsis could also contribute to insulin resistance in critically ill patients. Among the multitude of adipokines, this paper focuses on adiponectin, leptin, resistin, visfatin, chemerin, tumor necrosis factor-*α* (TNF-*α*), Interleukin (IL)-1, IL-6, IL-8, IL-10, plasminogen-aktivator-inhibitor-1 (PAI-1), monocyte chemoattractant protein-1 (MCP-1), and retinol-binding protein-4 (RBP-4) as adipokines with particular emphasis on their influence on severe sepsis and on sepsis-related insulin resistance.

## 2. Adiponectin

Adiponectin—the prototype of anti-inflammatory adipokines—is the most abundant adipokine produced almost exclusively by mature adipocytes. Its blood concentration is much higher than the concentration of other known hormones, accounting for approximately 0.01% of the total plasma protein [5]. Serum levels of adiponectin are diminished in obese individuals and correlate negatively with the degree of obesity (Table 1). Due to insulin-sensitizing effects of adiponectin in humans, its plasma levels are inversely correlated with insulin resistance in type 2 diabetes (T2D) [6]. Besides obesity, lower adiponectin levels have also been found in critically ill patients [3, 7]. For these patients, a strong association between plasma cortisol and adiponectin as well as an inverse correlation between plasma CRP and adiponectin has been described. Furthermore, adiponectin levels correlate negatively with severity of sepsis.

**Figure 1:**
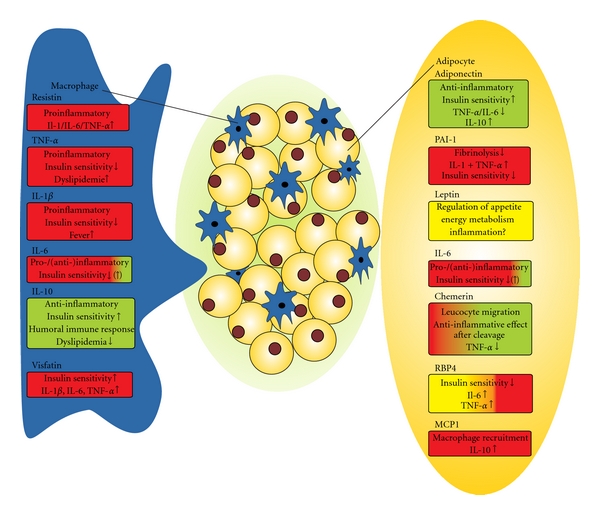
Primary cellular source of proinflammatory (red), anti-inflammatory (green), and neutral (yellow) adipokines.

Referring to the way of action, recent studies showed that adiponectin attenuates inflammation on several levels (Figure 1). It suppresses the function of mature macrophages and inhibits foam cell formation (lipid accumulation in macrophages) as well as growth of macrophage precursors [8]. In addition, adiponectin attenuates the production of TNF-*α* and IL-6 production in macrophages, induces that of anti-inflammatory IL-10, and inhibits Toll-like receptor family-induced signaling in mouse macrophages [9, 10]. Given the anti-inflammatory effects of adiponectin, it is plausible that lowered adiponectin levels may predispose to sepsis-related proinflammatory complications in states of obesity, diabetes, and insulin resistance. Further, the reduced adiponectin levels in septic patients may support insulin resistance in critically ill patients although it was shown, that adiponectin levels and insulin demand were positively correlated during sepsis [11].

## 3. Resistin

Besides adiponectin, resistin was reported to participate in the inflammatory response [12]. Resistin was originally discovered in mice as an adipocyte-derived hormone. It is increased in obese mice and causes insulin resistance in mice [13]. In contrast to mice, resistin in humans is mainly derived from macrophages rather than from adipocytes, so it is no surprise that proinflammatory resistin is elevated in a state of systemic inflammation [14]. Controversy exists on whether resistin can be considered a true adipose tissue-derived protein. Resistin is included, since serum resistin levels will increase with both increased adiposity and sever inflammation.

 Its secretion is stimulated by inflammatory processes, glucocorticoids, and lipopolysaccharides (LPSs), whereas TNF-*α* and *β*-adrenergic stimulation act as inhibitory [15]. Resistin increases transcriptional events leading to higher expression of several proinflammatory cytokines including IL-1, IL-6, IL-12, and TNF-*α* [16]. In a positive feedback loop, resistin can be upregulated by interleukins, and also by microbial antigens such as LPS [17]. In accordance with these reports, significantly higher resistin levels were found in septic patients, and resistin levels were associated with severity of sepsis supporting the hypothesis that resistin predominantly participates in systemic inflammatory response to infection [3]. No relationship between resistin concentration and insulin resistance has been found [18].

## 4. Leptin

The adipose-derived hormone leptin is well known for its contribution to energy metabolism and satiety signaling in the hypothalamus. Circulating leptin levels directly reflect adipose tissue mass. Furthermore, leptin affects glucose metabolism and increases insulin sensitivity. Obese humans are often insulin and leptin resistant [19]. The role of leptin in sepsis and septic shock is still controversially discussed. Earlier reports suggested that high lepin levels are associated with increased survival in sepsis and septic shock [20, 21], whereas several other reports fail to show a correlation between leptin and sepsis [22]. Minor changes of leptin serum levels in septic patients are reported with various slight increase or decrease during the course of sepsis, not being related either to survival or to metabolic and hormonal changes [23]. The influence of leptin on insulin resistance is still not fully understood, whereby studies in lipodystrophic patients and in patients with mutations of the insulin receptor have indicated that leptin therapy is associated with a marked improvement in the metabolic state of the patients with remarkable improvements in insulin sensitivity [24].

## 5. Plasminogen Activator Inhibitor-1 (PAI-1)

Plasminogen activator inhibitor-1 (PAI-1) is an inhibitor of fibrinolysis produced by visceral and subcutaneous adipocytes, endothelial cells, and stromal cells in visceral adipose tissue [25].

The plasminogen activation system is part of the fibrinolysis which is tightly regulated and protected against dysfunction by various activators and inhibitors. PAI-1 interacts with proteolytic mediators, including urokinase plasminogen activator. Microorganisms including bacteria have been proven to interact with components of the fibrinolytic pathways for their own benefits including dissemination within the host and evasion of host inflammatory immune response [26]. Studies in a rodent model suggested that microvascular thrombosis in sepsis is associated with inhibition of fibrinolytic processes by PAI-1 [27].

PAI-1 participates in acute inflammatory conditions with intrinsic proinflammatory properties via neutrophil activation and subsequent release of the proinflammatory cytokines IL-1 and TNF-*α* by neutrophils [28]. As in obesity, PAI-1 is elevated in inflammatory conditions, and serum levels correlate with the severity of sepsis [3]. Levels of PAI-1 are also positively related to poor outcome, increased severity of disease, and increased levels of various cytokines, acute-phase proteins, and coagulation parameters [29]. Elevated PAI-1 levels seem also to have a direct causal role in insulin resistance, since insulin sensitivity was enhanced significantly in obese mice lacking PAI-1 (high-fat/high-carbohydrate diet induced) [30].

## 6. Interleukins and TNF-***α***

Plasma concentrations of IL-1, IL-6, and IL-8, and TNF-*α* are increased in response to inflammation. Macrophages in adipose tissue are a significant source of these cytokines, whereby IL-6 and IL-8 are directly produced by adipocytes, in addition [31–34]. TNF-*α* and IL-1 are some of the most important mediators of inflammation [33]. IL-1 and TNF-*α* mutually enhance each other's production and act synergistically. Their expression and secretion increase upon obesity and correlate positively with the body mass index.

IL-1 and TNF-*α* are involved in obesity-related insulin resistance. IL-1 can promote *β*-cell destruction and alter insulin sensitivity and insulin signaling. TNF-*α* is known to promote lipolysis and the secretion of free fatty acids, which contribute to an increase in hepatic glucose production [35]. On a cellular level, TNF-*α* is a potent inhibitor of the insulin-stimulated tyrosine phosphorylation on the beta-chain of the insulin receptor and insulin receptor substrate-1 [36].

IL-6 can act both as a pro- and anti-inflammatory cytokines. It is known to act similarly to TNF-*α*, but controversial findings also have been reported for their actions on insulin sensitivity [37]. Acute IL-6 administration to humans increases insulin-stimulated glucose disposal and fatty acid oxidation *in vivo*. Furthermore, IL-6 stimulates the production of anti-inflammatory cytokines and suppresses TNF-*α* production in humans. In contrast, in adipose tissue, IL-6 concentration is inversely related to insulin-stimulated glucose transport in adipocytes and to insulin sensitivity [35]. Presumably, obesity and T2D-associated persistent systemic increases of IL-6 may trigger insulin resistance [38]. *In vitro* studies showed direct decreasing effects of IL-6 and TNF-*α* on adiponectin mRNA levels in adipose tissue cultures [39]. In analogy, elevated IL-1, IL-6, and TNF-*α* levels in septic patients may contribute to insulin resistance.

## 7. Chemerin

Chemerin is expressed in adipose tissue [40]. Initially, it was reported as a proinflammatory molecule, while later-on studies revealed the existence of an anti-inflammatory chemerin cleavage product [41]. It is synthesized as an inactive precursor (prochemerin) requiring the processing of its C terminus for generating active chemerin—an agonist of chemerin receptor 23 (ChemR23), a receptor expressed on dendritic cells and macrophages. Angiotensin-converting enzyme (ACE) may be responsible for the activation of prochemerin [42]. Receptor-ligand binding elicits chemotaxis of macrophages and immature, but not mature, dendritic cells [40]. In a mouse model of acute lung injury, chemerin displays potent anti-inflammatory properties, reducing neutrophil infiltration and inflammatory cytokine release [43]. Chemerin plasma levels have been shown to have a significant association with inflammation.

Since pro- and anti-inflammatory responses to a stimulus are often simultaneous—like a two-edged sword—chemerin was also shown to be proinflammatory by recruitment and retention of macrophages at sites of inflammation [44].

Chemerin levels increase with BMI in humans. Higher levels are associated with insulin resistance at the level of lipogenesis and insulin-induced antilipolysis in adipocytes. Chemerin also induces insulin resistance in human skeletal muscle cells [45]. Consequently, elevated secretion of chemerin may be involved in the negative crosstalk between adipose tissue and skeletal muscle contributing to the negative relationship between inflammation and insulin sensitivity.

## 8. Retinol Binding Protein-4 (RBP-4)

Retinol binding protein-4 (RBP-4) is the principal transport protein for retinol (vitamin A) produced by adipocytes and appears to affect insulin action. Increased serum RBP-4 levels have been reported in obesity and T2D and decrease with improved insulin sensitivity [46]. In mice, transgenic expression of RBP-4 caused insulin resistance, and RBP-4 knockout mice display enhanced insulin sensitivity [46]. Oversecretion of RBP-4 may negatively affect beta-cell function directly or by preventing the binding of transthyretin—a *β*-cell stimulus—to its receptor [47]. Furthermore, RBP-4-associated effects are the increase of hepatic gluconeogenesis by enhancing the expression of phosphoenolpyruvate carboxykinase in the liver and the attenuated insulin signaling in skeletal muscle [46].

RBP-4 is associated with altered insulin resistance in critical illness. Patients with sepsis had lower levels of RBP-4 than did nonseptic patients. When critical illness was sustained, RBP-4 levels returned to normal reference values. Insulin therapy blunts the rise of RBP-4 levels [48]. In acute critically ill patients, serum RBP-4 concentrations were significantly reduced and showed a strong association with hepatic dysfunction, insulin resistance, and acute mortality, possibly as a negative acute phase reactant [49]. RBP-4 can induce the production of IL-6 and TNF-*α* in bone marrow derived macrophages [50].

## 9. Visfatin

Visfatin—also known as pre-B-cell-enhancing factor (PBEF)/nicotinamide phosphoribosyl transferase (NAMPT)—was named for the suggestion that it would predominantly be produced and secreted in visceral fat. However, it was found to be expressed fairly widely by various cell types throughout the body, in particular from macrophages in response to inflammatory signals rather from adipocytes [51]. PBEF/NAMPT/visfatin serves intracellularly as the rate-limiting enzyme that catalyzes the first step in nicotinamide adenine dinucleotide (NAD) biosynthesis from nicotinamide and that regulates growth, apoptosis, and angiogenesis of mammalian cells [52]. Extracellular PBEF/NAMPT/visfatin is reported to exert insulin-mimetic effects in cultured cells and to decrease plasma glucose levels in mice by binding to and activating the insulin receptor. However, the physiological relevance of visfatin remains controversial due to conflicting results regarding its possible connection to obesity, T2D, and other metabolic complications in subsequent studies [53].

Clinical and experimental studies have shown that the expression and secretion of PBEF/NAMPT/visfatin is upregulated during inflammation and in response to proinflammatory cytokines. Furthermore, PBEF/NAMPT/visfatin itself can contribute to the inflammatory processes by triggering cytokine production and NF-kappaB activation [54]. It is expressed at high levels in neutrophils harvested from septic critically ill patients and contributes to prolonged neutrophil survival in clinical sepsis [55]. It exerts proinflammatory properties in activating human leukocytes and introduces IL-1*β*, TNF-*α*, and especially IL-6 production [56]. Biochemical neutralization of PBEF/NAMPT/visfatin has been proven to be effective in several models of inflammation including sepsis [57].

## 10. MCP1

Monocyte chemotactic protein-1 (MCP-1)—also known as CCL-2 (CC-chemokine ligand 2)—produced by adipocytes contributes to macrophage infiltration in adipose tissue [58]. Chemokines are known to play an important role in the pathogenesis of sepsis and endotoxemia. Increased concentrations of MCP-1 have been associated with sepsis and prediction of sepsis-related mortality [59].

Its pathophysiological role has been linked to the activated protein C pathway and its induced genes [60]. MCP-1 positively regulates IL-10 but negatively controls macrophage migration inhibitory factor (MIF) in experimental peritoneal sepsis, suggesting an important immunomodulatory role for MCP-1 in controlling the balance between proinflammatory and anti-inflammatory factors in sepsis [61]. Serum levels of MCP-1 in septic patients are increased considerably compared to a much lower rise in the morbidly obese. These acute increased serum concentrations of MCP-1 in sepsis seem to contribute substantially to systemic insulin resistance—irrespective of a preexisting adipose tissue inflammation [62].

## 11. Conclusions

Adipokines reveal proinflammatory and/or anti-inflammatory effects. Adiponectin—the prototype of an anti-inflammatory adipokines and the most abundant adipokine—is diminished in sepsis, while the levels of resistin—a protein with proinflammatory properties—are elevated. Most other adipokines in humans are either increased or decreased in sepsis, and alterations are in the same directions as in obese individuals. In obesity, chronic inflammation in adipose tissue is thought to be important for the development of insulin resistance. Hyperglycemia and insulin resistance are also well-known features of critical illness. The finding of altered levels of adipokines in septic patients underlines its possible contribution in the development of hyperglycemia and insulin resistance in these patients.

## Figures and Tables

**Table 1:** Proinflammatory or anti-inflammatory properties of adipokines, serum level changes of adipokines in septic or obese patients, and influence of adipokines on insulin sensitivity in patients with sepsis or obese patients.

Adipokine	Anti-	Pro-	Humans with		Insulin
	inflammatory effect		sepsis	obesity	sensitivity
Adiponectin	+		↓	↓	↑
Chemerin	+		↑	↑	↓
Resistin		+	↑	→	→
PAI-1		+	↑	↑	↓
Visfatin		+	↑	↑	
MCP-1		+	↑	↑	↓
TNF-*α*		+	↑	→ (?)	↓
IL-1		+	↑	↑	↓
IL-6		+	↑	↑	↓
IL-8		+	↑	↑	↓
IL-10	+		↑	↑	↑
Leptin	→	→	→	↑	?
RBP 4		+	↓	↑	↓
